# Big data analytics frameworks for the influence of gut microbiota on the development of tic disorder

**DOI:** 10.3389/fncom.2022.986591

**Published:** 2022-08-25

**Authors:** Fei Fan, Zhaoxiang Bian, Xuan Zhang, Hongwei Wu, Simeng Wang, Si Zhang, Qiong Wang, Fei Han

**Affiliations:** ^1^Department of Pediatrics, Guang’anmen Hospital, China Academy of Chinese Medical Sciences, Beijing, China; ^2^Chinese EQUATOR Centre, Hong Kong Chinese Medicine Clinical Study Centre, Chinese Clinical Trial Registry (Hong Kong), School of Chinese Medicine, Hong Kong Baptist University, Kowloon, Hong Kong SAR, China; ^3^Institute of Chinese Materia Medica, China Academy of Chinese Medical Sciences, Beijing, China; ^4^Clinical Medical School, Beijing University of Chinese Medicine, Beijing, China

**Keywords:** tic disorder, gut microbiota, data analysis, bacteroidetes, firmicutes

## Abstract

The association between gut microbiota and psychiatric disorders has received increasing research attention. Meanwhile, big data analysis has been utilized in many filed including business, human healthcare analysis, *etc*. The primary objective of this article was to provide insights into Big Data Analytics (BDA) to clarify the association between gut microbiota and TD (Tic disorder). Specifically, we investigated the recent studies related to gut microbiota composition differences in patients with TD compared to health people. We searched on PubMed and Embase (Ovid) databases for relevant published articles until June 15, 2021. A total of 78 TD and 62 health control stool samples were examined. Case-control design was applied in all the studies. No consensus was evident in α-diversity and β-diversity. The abundance of phyla *Bacteroidetes* and *Firmicutes* was predominant at the taxa level. Gut microbiota taxonomic differences were found between TD cases and controls, though inconsistently across studies. Further studies are needed to reveal the underlying pathophysiology of TD and correlation between TD and gut microbiota composition.

## Introduction

Tic disorder (TD) is characterized by sudden, recurrent, non-rhythmic movement, or phonic tic with childhood onset, ongoing throughout adulthood (Plessen, 2013). According to the Diagnostic and Statistical Manual of Mental Disorders (DSM)-5 (American Psychiatric Association., 2013), TD includes Tourette syndrome (TS), chronic motor or vocal tic disorder (CTD), provisional tic disorder (PTD), other specified tonic disorders, and unspecified tic disorders. TD is the most common movement disorder in children, but the reported prevalence of TD varies considerably (Cubo et al., 2011; Yang et al., 2016; Mohammadi et al., 2021) because a significant proportion of patients do not recognize their tics (Ueda and Black, 2021). Children with TD may experience subjective discomfort, sustained social problems, sleep difficulties, and many emotional problems (Conte et al., 2020; Fernández de la Cruz and Mataix-Cols, 2020; Isaacs et al., 2021). TD is commonly associated with obsessive-compulsive disorder (OCD), attention-deficit/hyperactivity disorder (ADHD), and anxiety disorders (Hirschtritt et al., 2015; Eapen et al., 2016). Thus, research to understand the development of TD is receiving increasing attention lately. TD occurs through interactions including but not limited to genetic (Cao et al., 2021), neurobiochemical (Kanaan et al., 2017), inflammation-related (Martino et al., 2021), immunological (Lamothe et al., 2021), and environmental factors (Storch et al., 2017). However, its pathophysiology remains unknown.

Gut microbiota is a variety of microorganisms in the gastrointestinal tract, normally more than 1,000 bacterial species and with more than nine million genes. Gut microbiota is extremely diverse and changeable with the majority of bacteria from the four dominant phyla including Bacteroides, Firmicutes, Proteobacteria, and Actinobacteria, which constitutes more than 98% of all of the human gut microbes. Gut microbiota constitute a very important part in both of the health maintenance and the disease pathogenesis process. It is a known fact that a diverse and stable and gut microbiota is essential to for various normal physiologic functions such as immunology regulation, prevention of bacterial infection, energy harvest and metabolism, and so on. Meanwhile, the gut microbiota is associated with disease is often characterized by a decrease or increase in species richness and proliferation of some specific pathogens. The gut microbiota plays an important role in the extensive reciprocal connections between the gastrointestinal system and human brain, forming the microbiome-gut-brain axis (Cryan et al., 2020). The association between gut microbiota and psychiatric disorders has received increasing research attention (Morais et al., 2021). Over the past decade, many studies have revealed that the gut microbiota is directly involved in the production of various neurotransmitters, such as gamma-aminobutyric acid (GABA), serotonin (5-HT), glutamate, and dopamine (DA) (Bull-Larsen and Mohajeri, 2019; Altaib et al., 2021; Bhatt et al., 2022), which are closely associated with a number of psychiatric disorders, including TD (Kanaan et al., 2017), ADHD (Turna et al., 2020), OCD (Simpson et al., 2021), and anxiety (Ridaura and Belkaid, 2015).

Gastrointestinal symptoms are not common in TD patients (Fernández de la Cruz and Mataix-Cols, 2020). However, studies show that TD patients have a higher risk of metabolic or cardiovascular disease than the general population, which also plays an important role in the pathogenesis and course of TD, suggesting a relationship between TD and microbiota (Brander et al., 2019; Fernández de la Cruz and Mataix-Cols, 2020; Tomasova et al., 2021). Most TD patients have sleep disorder (Hibberd et al., 2020; Isomura et al., 2022) and are sensitive to psychological stress (Tilling and Cavanna, 2020). Meanwhile, gut microbiota can get disrupted under psychological stress (Madison and Kiecolt-Glaser, 2019; McGuinness et al., 2022) and is correlated with the sleep behavior (Qi et al., 2022). Recent studies have shown that the gut microbiota plays an indispensable role in regulating microglial maturation and function (Bairamian et al., 2022). Circulation of microbe-derived neurotransmitters, including acetylcholine, GABA, and 5-HT, can regulate microglial activation (Fung et al., 2017). Interestingly, abnormalities in microglial activation, development, and function in the basal ganglia of TD patients are also widely recognized (Frick and Pittenger, 2016). Some studies have demonstrated that fecal microbiota transplantation (FMT) effectively ameliorates TD symptoms (Zhao et al., 2017, 2020). Animal studies have also shown that microbiota have the potential to improve tic syndromes (Liao et al., 2019). Despite evidence pointing to a connection between gut microbiota and TD, the nature of this relationship remains unclear. Better understanding of which microbiome is associated with TD and its pathophysiological effects will enable researchers to provide new therapeutic and diagnostic avenues of TD in the future.

Thus, the primary objective of this review was to investigate and compare the recent studies relating to gut microbiota composition differences in patients with TD.

Thus, the primary objective of this work is to summarize, investigate and compare recent studies on gut microbiota composition differences in patients with TD.

## Materials and methods

This work has been uploaded and accepted into PROSPERO under the identification number CRD42021265088, performed in accordance with PRISMA guidelines (Page et al., 2021).

### Information sources

The databases PubMed and Embase (Ovid) were searched for human studies in English up until June 15, 2021, using the following search strategies (for PubMed): [”tic disorder”(Text Word) OR “tic disorders”(Text Word) OR “tourette syndrome”(Text Word) OR “gilles de la tourette”(Text Word) OR “pediatric autoimmune neuropsychiatric disorders associated with streptococcal infections”(Text Word)] AND [”gut microbiota*”(Text Word) OR “gut microbiome*”(Text Word) OR “intestinal microbiota”(Text Word) OR “intestinal microbiome”(Text Word) OR “gastrointestinal microbiota”(Text Word) OR “gastrointestinal microbiome”(Text Word)] (Supplementary Material 1). Gray literature was included if fulfill the inclusion criteria.

### Inclusion and exclusion criteria

Inclusion criteria:

• Original observational studies performed on TD patients diagnosed according to DSM-5 (or IV) or ICD-11 (or 10).
• Detection of gut microbiota composition through high-throughput sequencing techniques.
• Inclusion of a healthy control (HC) group.
• Published in English.

Exclusion criteria:

• Animal studies.

### Study selection

Studies were imported into the Mendeley reference manager^1^ to remove duplicates using its automatic function. Files generated from PubMed and Embase were reviewed and selected using the website: http://syrf.org.uk independently by authors FF and SW based on titles and abstracts, and later the included studies were whole-text reviewed manually. Studies inconsistently agreed upon both reviewers were resolved by a third author, FH.

### Outcome measures

Data were extracted from the TD and HC groups using a Microsoft Excel file (Supporting Information 2), focusing on the demographics, microbiota analysis methodology, α- and β-diversity, clinical information, and other relevant findings. A meta-analysis was not performed in the present study.

### Risk of bias assessment

The Newcastle-Ottawa Scale (NOS) was used to evaluate the risk of bias in case–control studies. The NOS scale contains three categories comprising total of eight items: selection (four items), comparability (one item), and exposure (three items). Quality score with a maximum of ten was obtained using a rating algorithm: 0–5 (poor), 6–7 (moderate), and 8–10 (high).

## Results

### Study selection

**FIGURE 1:**
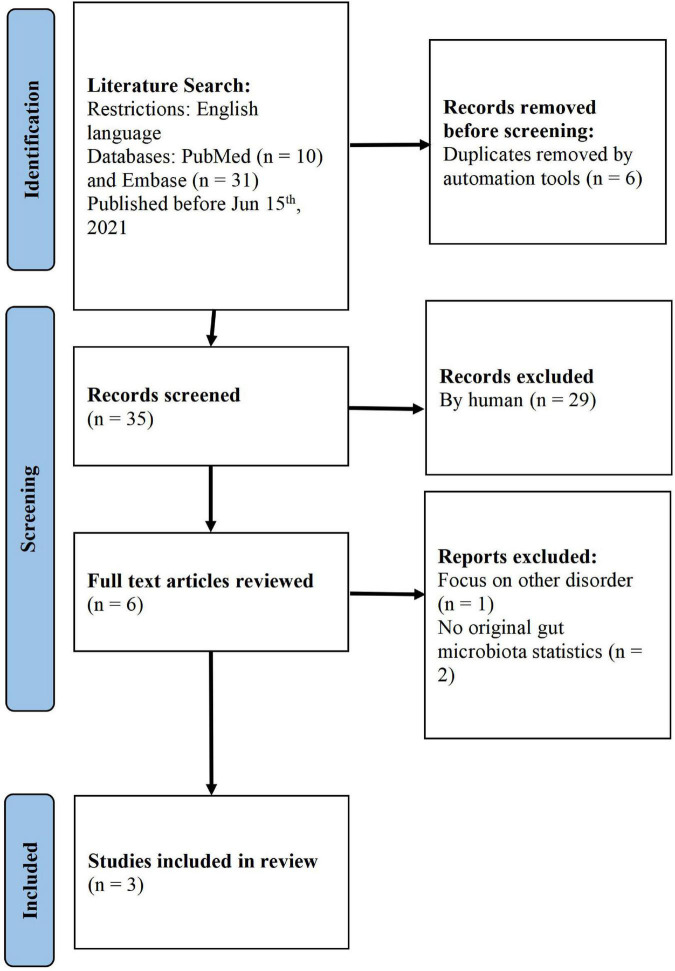
PRISMA flowchart of the screening process.

Study selection was conducted using the PRISMA guidelines. Using keywords, we found 41 studies from the literature search. After the automatic removal of duplicates, 35 unique articles were identified. After screening the titles and abstracts of these articles, six were assigned to a full-text assessment, out of which three unqualified articles were removed (one did not focus on TD and two did not have original gut microbiota statistics). Finally, we focused on three articles for further analysis (Lee and Wong, 2018; Zhao et al., 2020; Xi et al., 2021; Figure 1).

### Assessment of study quality/bias

Estimates of bias were obtained for the three studies that compared patients with TD with HCs using the NOS, as indicated in Table 1. One study (Xi et al., 2021) received a score of six (moderate) because the interview was not blinded to the status. The second study received a score of four (low) (Zhao et al., 2020) due to the HC being only one child and thus the resulting potential biases, and the last study received three (low) (Lee and Wong, 2018) due to inadequate description of the study.

**TABLE 1:** Quality assessment of included studies based on Newcastle-Ottawa scale (NOS).

No.	Study	Year	Selection	Comparability	Exposure	Total
1	Lee and Wong (2018)	2018	1	1	1	3
2	Zhao et al. (2020)	2020	2	1	1	4
3	Xi et al. (2021)	2021	3	2	1	6

### Characteristics of studies

Demographic data of the three studies are shown in Table 2. Two out of three studies were conducted in Beijing, including a total of 54 patients diagnosed with TD and 51 HCs (Zhao et al., 2020; Xi et al., 2021). The other study was conducted in Taiwan, which included 24 TD patients and 11 HCs (Lee and Wong, 2018). The total sample size of the selected studies ranged from 6 to 99, with the number of cases ranging from 5 to 49, and the number of controls ranging from 1 to 50. With these three studies combined, a total of 78 cases and 62 controls were investigated and included TD patients and HCs younger than 18 years. Moreover, the study design of two studies was cross-sectional and compared gut microbiota in TD patients with that in a HC group (Lee and Wong, 2018; Xi et al., 2021).

**TABLE 2:** Demographic data of the studies.*^a^*

No.	Study	Year	City	Participants		Age mean (SD)		Male (m/f)		BMI mean (SD)	
							
				TD	HC	TD	HC	TD	HC	TD	HC
1	Lee and Wong (2018)	2018	Taiwan	*n* = 24	*n* = 11	NA	NA	NA	NA	NA	NA
2	Zhao et al. (2020)	2020	Beijing	*n* = 5	*n* = 1	8	14	5/0	1/0	18.0	NA
3	Xi et al. (2021)	2021	Beijing	*n* = 49	*n* = 50	8.84 (2.35)	8.78 (2.26)	38/11	39/11	18.28 (2.99)	17.22 (2.66)

^a^Data are presented as mean (standard deviation, SD) or number of participants. m, male; f, female; TD, tic disorder; HC, healthy controls; BMI, body mass index; DSM-5, Diagnostic and Statistical Manual of Mental Disorders-5.

In two studies (Zhao et al., 2020; Xi et al., 2021), patients were assessed according to the DSM-5 criteria. We found that only one study (Xi et al., 2021) mentioned gastrointestinal disturbances (mild constipation and abdominal pain), and provided gastrointestinal severity index (GSI) scores. Two studies (Zhao et al., 2020; Xi et al., 2021) included cases that received dopamine receptor antagonists (DRA) and other medications, while the rest (Zhao et al., 2020; Xi et al., 2021) did not mention these criteria. In addition, only one study (Xi et al., 2021) excluded antibiotics/probiotics taken within 4 weeks prior to sample collection and any infective or other severe disease conditions that may influence the gut microbiota. The ability to compare or interpretation of individual studies is limited by the extensive variability of different aspects of the studies (Table 3).

**TABLE 3:** Clinical information of patients with tic disorder (TD) and healthy controls (HCs).*^a^*

No.	Study	Diagnoses (n)	Diagnostic instrument	Disease duration (SD), year	YGTSS scores (SD)	Comorbidities (n)	GSI (SD)	Gastrointestinal disturbances (%)	Medication (n)
1	Lee and Wong (2018)	TS: severe tics (14); mild tics (10)	N/A	severe tics: 4.5 (2.33) mild tics: 2.25 (2.5)	TTS scores: severe tics, 27.4 (7.5); mild tics, 14.8 (4.1)	N/A	N/A	N/A	N/A
2	Zhao et al. (2020)	TS	DSM-5	1.5–4	YGTSS-TTS > 13	ADHD (3), variant asthma (1)	N/A	N/A	Tiapride (3); aripiprazole (2); trihexyphenidyl (2); risperidone (1)
3	Xi et al. (2021)	TD: TS (23); PTD (17); CTD (9)	DSM-5	2.11 (1.92)	36.71 (16.73)	N/A	2.31 (1.86)	mild constipation, 26.53; abdominal pain, 28.57	DRAs (12); topiramate (1); valproate (1); treatment-naive (35)

^a^SD, standard deviation; YGTSS, Yale Global Tic Severity Scale; YGTSS-TTS, Yale Global Tic Severity Scale Total Tic Scale (combined motor tic and vocal tic score); GSI, Gastrointestinal Severity Index; TD, tic disorder; PTD, provisional tic disorder; CTD, chronic motor or vocal tic disorder; TS, Tourette syndrome; DRA, dopamine receptor antagonist.

### Microbiota analysis

There were some differences in the sample analysis with respect to the diversity of results in the included studies, as shown in Table 4. Two out of three studies (Zhao et al., 2020; Xi et al., 2021) used shotgun metagenomic sequencing and analyzed the α-diversity and β-diversity of their samples without mentioning the exact index.

**TABLE 4:** Microbiota analysis methodology and diversity results.*^a^*

No.	Study	Samples	Stool storage	Genetic quantification	Alpha diversity	Beta diversity
1	Lee and Wong (2018)	Stool	N/A	N/A	N/A	N/A
2	Zhao et al. (2020)	Stool	−80°C	Shotgun metagenomic sequencing	A reduced OTU number	A different cluster in PCoA
3	Xi et al. (2021)	Stool	−80°C	Shotgun metagenomic sequencing	No significant difference*^b^*	No significant difference*^b^*

^a^OTU, operational taxonomic unit; TD, tic disorder; HC, healthy controls; PCoA, principal coordinate analysis. ^b^Between treatment-naïve TD patients and HCs.

### Microbiota findings

The gut microbiota of TD patients was compared to that of HCs to assess changes in different individuals’ bacterial abundances. The findings are presented in Table 5 and a more comprehensive listing in Supplementary Material 2. A study by Lee and Wong (2018) stated that the *Prevotellaceae* family and *Prevotella* genus were decreased and *Ruminococcus* genus was increased in TD patients. In the study by Zhao et al. (2020), *Bifidobacterium*, *Catenibacterium*, *Collinsella*, and *Dorea* genera were decreased in TD patients. In another study by Xi et al. (2021), the species *Bacteroides plebeius*, *Ruminococcus lactaris*, *Prevotella* stercorea, and *Streptococcus lutetiensis* were decreased in TD patients. Moreover, Xi et al. (2021) found that *Bacteroides eggerthii*, *Bacteroides dorei*, and *Bacteroides thetaiotaomicron* species were positively correlated with the Yale Global Tic Severity Scale (YGTSS) scores (as with the severity of tics). Genus *Prevotella* was negatively correlated with the severity of tics in another study (Lee and Wong, 2018).

**TABLE 5:** Different microbiota findings in tic disorder (TD) patients.*^a^*

No.	Study	Gut microbiota profiles	Other findings
1	Lee and Wong (2018)	Family:	*Prevotella* was negatively correlated with the severity of tics.
		↓:*Prevotellaceae*	
		Genus:	
		↑:*Ruminococcus*	
		↓:*Prevotella^b^*	
		Species:	
		↓:*Clostridium bartlettii*, *Prevotella copri*, and *Subdoligranulum variabile*	
2	Zhao et al. (2020)	Genus:	
		↓:*Bifidobacterium*, *Catenibacterium*, *Collinsella*, and *Dorea*	
		Species:	
		↑:*Bacteroides vulgatus*	
		↓:*Allisonella histaminiformans*, *Bacteroides coprocola*, *Catenibacterium mitsuokai*, *Dialister succinatiphilus*, *Holdemanella biformis*, and *Roseburia faecis*	
3	Xi et al. (2021)	Species: ↑:*Bacteroides plebeius*, *Ruminococcus lactaris* ↓:*Prevotella stercorea*, *Streptococcus lutetiensis*	*Bacteroides eggerthii*, *Bacteroides dorei*, and *Bacteroides thetaiotaomicron* positive correlations with the YGTSS scores.

^a^TD, tic disorder; YGTSS, Yale Global Tic Severity Scale. ^b^Severe TS samples (n = 14).

## Discussion

Due to the limited treatment methods for tic disorder at present, and the effectiveness of some treatment methods is not so effective, or the effectiveness is limited, so the exploration of its pathogenesis is particularly important, which will guide the better diagnosis and treatment of tic disorder in the future. In recent 10 years, in addition to finding better drug treatments, there are more and more studies on the influences of both hereditary and environmental factors on the occurrence and development of tic disorders (TD). Understanding the microbiome associated with TD has the potential to further research on TD pathophysiology and provide individual treatment options. Although many microbiome infections appear to be correlated with TD (Müller et al., 2004; Mell et al., 2005; Prasad, 2021), to our knowledge, so far no study has revealed the fine-grain pathophysiology. In this work, we attempt to assess whether individuals with TD had a distinct gut microbiota composition compared to HCs. Notably, all the studies identified that the gut microbiota of individuals with TD were distinguishable from that of HCs, although the results of each study varied. The fine structure of the gut microbiota varies greatly among cases (Caporaso et al., 2011).

### Main findings

Overall, no consensus regarding α-diversity and β-diversity was found. Xi et al. (2021) found no significant differences in diversity. However, Zhao et al. (2020) found some possible differences, but this was not described in detail. At the taxa level, the abundance of phyla *Bacteroidetes* and *Firmicutes* was the predominant difference between TD patients and HCs. One family, one genus, and three species of *Bacteroidetes* were found to be decreased, while two species were found to be increased in patients with TD. Two genera and eight species of *Firmicutes* were found to be decreased, while one genus and one species were found to be increased in TD patients. A study by Lee and Wong (2018) found that the proportion of genus *Prevotella* was negatively correlated with the severity of tics. Meanwhile, Xi et al. (2021) found that the species *Bacteroides eggerthii*, *Bacteroides dorei*, and *Bacteroides thetaiotaomicron* were positively correlated with severity. *Bacteroidetes* and *Firmicutes* phyla are also the most dominant gut microbiota in normal people (Jandhyala et al., 2015) and are correlated with inflammatory conditions such as inflammatory bowel disease (Stojanov et al., 2020). The establishment of the gut microbiota has been shown to be a progressive process, and the ratio of *Firmicutes* to *Bacteroidetes* is significantly correlated with human age (Ley et al., 2006). The *Firmicutes*/*Bacteroidetes* ratio increases from birth to adulthood and further changes with age (Mariat et al., 2009). Reports have shown that changes in the ratio of *Firmicutes*/*Bacteroidetes* are significant factors affecting childhood diseases childhood obesity (Indiani CMDSP et al., 2018), autism spectrum disorders (ASD) (Strati et al., 2017), and others (Quagliariello et al., 2016; Valentini et al., 2020). TD typically begins in childhood and often improves in early adulthood, but the reason remains unknown (Hartmann et al., 2020). Current studies link age correlation with TD and the ratio of *Firmicutes* to *Bacteroidetes*, although the result is still not definitive. Further studies should focus on this ratio to reveal more comparable results.

Bacteria with increased abundance were found in the gut microbiota of patients with various inflammatory diseases (Zhang et al., 2015; Mondot et al., 2016), suggesting a potential pro-inflammatory effect. Moreover, other studies suggest that decreased abundance of genus *Bifidobacterium* (Plaza-Díaz et al., 2017) and species *Holdemanella biformis* (Pujo et al., 2021), which also decreased in this study, had an anti-inflammatory effect. Zhao et al. (2020) analyzed a wide range of inflammatory markers associated with the gut microbiota. Several studies have confirmed this mechanism, and reported elevated levels of pro-inflammatory cytokines [including IL-12 and TNF-α (Leckman et al., 2005)] and decreased levels of anti-inflammatory cytokines (including IL-13) in TD patients (Parker-Athill et al., 2015). In addition, the decreased levels of *Prevotella copri*, *Prevotella stercorea*, and *Roseburia faecis* also determine short-chain fatty acid (SCFA) levels (Louis et al., 2010; Liu et al., 2021). SCFAs play an anti-inflammatory and antimicrobial role in various interactions between gut microbiome and host metabolism (Tan et al., 2014; Sanna et al., 2019). Additionally, microbial metabolites can affect central neurotransmitters by activating afferent nerve fibers. SCFAs can stimulate the release of central neurotransmitters (including 5-HT) in the intestine (Yano et al., 2015). *Bifidobacterium* is a key member of the human gut microbiota affecting GABA production (Barrett et al., 2012). High levels of *Ruminococcus lactaris* (Dan et al., 2020) and low levels of the genera *Collinsella* and *Dorea* (Strati et al., 2017) have also been found in ASD patients with constipation symptoms, further explaining the potential role and related symptoms of *Ruminococcus lactaris* in the pathological mechanism of neurodevelopmental disorders.

### Treatment and diet

Although there have studies that attempted to utilize FMT (Zhao et al., 2017, 2020) in the treatment of TS (the most severe type of TD), the results have been limited. Zhao et al. (2020) found that FMT might reduce fecal lipopolysaccharide levels in TD patients and increase *Bacteroides coprocola* and *Dialister succinatiphilus* abundance and decrease *Bacteroides vulgatus* abundance. In the study by Xi et al. (2021), DRA-treated patients showed enrichment of *Bacteroides dorei*, *Escherichia coli*, *Bacteroides caccae*, and *Ruminococcus gnavus*. These enterotypes also seem to have some functional relevance to diet. The genus *Bacteroides* is associated with high-fat or high-protein diets and *Prevotella* with high-carbohydrate diets (Wu and Hui, 2011).

### Risk of bias

Of the three studies, Xi et al. (2021) displayed age and BMI information as mean and SD, and Zhao et al. (2020) included mean age and BMI. It has been reported that age and BMI are related to the composition of the gut microbiota (Haro et al., 2016; Odamaki et al., 2016). The study by Zhao et al. (2020) was the only study with all-male cases. This actually made the samples more homogeneous because gut microbiota composition has also been shown to differ according to sex (Haro et al., 2016). Lee and Wong (2018) study had scarce demographic data. Although all included studies reported YGTSS scores, there was a lack of consistent diagnostic criteria for the case definition. The reliability and accuracy of microbiome studies depend largely on the molecular biology techniques used, and differences in databases can affect the results of microbiome data (Haro et al., 2016). The studies in this review lack such information, and it is recommended that all studies use uniform classification criteria and databases to obtain more comparable results.

### Limitation

However, there are several limitations that should be acknowledged. First, this review included only three studies and a small sample size; thus, more TD patients enrolled from different studies are needed to make our results more reliable and reasonable. Second, *in vitro* and *in vivo* experiments were not conducted in the included studies. Finally, differences in the study population, including age, sex, height, weight, genetics, emotion, stress, and environmental factors, were not analyzed in the included studies.

## Conclusion

Emerging scientific data support the significant role of the gut microbiota in the regulation of the central nervous system. The results of the included studies show that the gut microbiota in children with TD is significantly different from healthy children. There is variability in microbial diversity as well as the abundance of taxa in patients with TD, which suggesting the complicity of the phenomenon. Furthermore, pro-inflammatory cytokines and central neurotransmitters may both play an important role in the pathophysiology of the gut microbiota in TD.

## Data availability statement

The original contributions presented in this study are included in the article/Supplementary material, further inquiries can be directed to the corresponding authors.

## Author contributions

FF and SW contributed to the study conception. HW designed the project. ZB and XZ collected the data and performed the formal analysis of finding. QW and SZ organized and integrated the data. FF drafted the manuscript. FF and FH critically reviewed the manuscript. ZB contributed to the visualization. FH acquired the funding source. All authors have read and agreed to the published version of the manuscript.

## Footnotes

1
https://www.mendeley.com/

## Supplementary material

Supplementary Material 1 Methodology.

Supplementary Material 2 Microbiota analysis.

Supplementary Material 3 Study quality of case-control studies.

## Abbreviations

TD: Tic disorder
DSM: Diagnostic and Statistical Manual of Mental Disorders
TS: Tourette syndrome
CTD: chronic motor or vocal tic disorder
PTD: provisional tic disorder
OCD: obsessive-compulsive disorder
ADHD: attention-deficit/hyperactivity disorder
GABA: gammaaminobutyric acid
5-HT: serotonin
DA: glutamate and dopamine
FMT: fecal microbiota transplantation
HC: healthy control
NOS: Newcastle-Ottawa Scale
GSI: gastrointestinal severity index
DRA: dopamine receptor antagonists
YGTSS: Yale Global Tic Severity Scale
ASD: autism spectrum disorders
SCFA: short-chain fatty acid.
