# Supplementary material for: Big data analytics frameworks for the influence of gut microbiota on the development of tic disorder
Source: Front Comput Neurosci. 2022 Aug 25;16:986591. doi: 10.3389/fncom.2022.986591 (PMC9452719; doi:10.3389/fncom.2022.986591)
Supplement: Supplementary Material 1 — Methodology. [file Data_Sheet_1.docx]

# **Supplementary Material 1**

# **Methodology**

**1. The research will be structured based on the PICO strategy:**

 √P (Population): Tic disorders(TD)

 √I (Intervention): Gut microbiota composition

 √C (comparison): Health control

 √O (Results): Describe the association between the gut microbiota and tic disorders

**2. Eligibility criteria:**

 √Articles published up until Jun 15th, 2021

 √Studies published in English

**3. Exclusion criteria:**

 √Articles that carried out experiments on animals.

1. **Databases search terms**

**Use of “AND” or “OR” in the search engines has been indicated.**

|  | Horizontal lines divided by “AND” | |
| --- | --- | --- |
|  | Cases | Outcome |
| Search terms (Vertical lines divided by “OR”) | Tic disorders[Text word] | Gut microbiota[Text Word] |
|  | Tic disorder[Text word] | Gut microbiome[Text word] |
|  | Gilles de la Tourette[Text word] | Intestinal Microbiota[text word] |
|  | Tourette syndrome[Text word] | Intestinal Microbiome[text word] |
|  | Pediatric autoimmune neuropsychiatric disorders associated with streptococcal infections[Text word] | Gastrointestinal microbiota[Text word] |
|  |  | Gastrointestinal microbiome[Text word] |
